# Postoperative pain after MiniLap percutaneous versus standard laparoscopic salpingo‐oophorectomy: A propensity‐matched study

**DOI:** 10.1002/ijgo.70060

**Published:** 2025-03-11

**Authors:** Stefano Restaino, Federico Paparcura, Martina Arcieri, Alice Poli, Giulia Pellecchia, Giorgio Bogani, Cristina Taliento, Luigi Della Corte, Federica Perelli, Jvan Casarin, Salvatore Gueli Alletti, Tiziana Bove, Teresa Dogareschi, Lorenza Driul, Francesco Fanfani, Anna Fagotti, Giovanni Scambia, Giuseppe Vizzielli

**Affiliations:** ^1^ Department of Maternal and Child Health, Obstetrics and Gynecology Clinic University Hospital of Udine Udine Italy; ^2^ PhD School in Biomedical Sciences, Gender Medicine, Child and Women Health University of Sassari Sassari Italy; ^3^ Department of Medicine (DMED) University of Udine Udine Italy; ^4^ Department Oncologic Surgery, Gynecologic Oncology Unit Fondazione IRCCS Istituto Nazionale Dei Tumori di Milano Milan Italy; ^5^ Department of Gynecological, Obstetrical and Urological Sciences ‘Sapienza’ University of Rome Rome Italy; ^6^ Maternal and Child Department, Unit of Obstetrics and Gynecology S. Anna University Hospital Cona Ferrara Italy; ^7^ Department of Neuroscience, Reproductive Sciences and Dentistry, School of Medicine University of Naples Federico II Naples Italy; ^8^ Department of Gynecology and Pediatrics Azienda USL Toscana Centro Florence Italy; ^9^ Department of Obstetrics and Gynecology, ‘Filippo Del Ponte’ Hospital University of Insubria Varese Italy; ^10^ Department of Obstetrics and Gynecology Ospedale Buccheri La Ferla—Fatebenefratelli Palermo Italy; ^11^ Department of Anesthesia and Intensive Care Medicine ASUFC University Hospital of Udine Udine Italy; ^12^ Department of Woman, Child Health and Public Health Fondazione Policlinico Universitario A. Gemelli IRCCS Rome Italy; ^13^ Universita' Cattolica del Sacro Cuore Rome Italy

**Keywords:** gynecologic surgery, MiniLap, percutaneous surgical system, salpingo‐oophorectomy

## Abstract

**Objective:**

The aim of this study was to compare postoperative outcomes in patients undergoing standard laparoscopic salpingo‐oophorectomy versus those using the MiniLap percutaneous surgical system, aiming to demonstrate the non‐inferiority of this ultra‐minimally invasive surgical technique compared to the current gold standard.

**Methods:**

This was a retrospective, single‐center propensity‐matched case–control study. A total of 80 surgical patients undergoing salpingo‐oophorectomy for benign pathology were selected, with 40 in each group (MiniLap in group A and S‐LPS in group B). Postoperative pain was subjectively reported at 2, 4, 12, and 24 h after surgery. The secondary outcomes of this study were to evaluate differences in the duration of surgery, intraoperative blood loss, postoperative complications, and cosmetic results.

**Results:**

The median operative time was 57.7 min (range, 28–125) in group A and 75.5 min (range, 22–180) in group B (*P* value 0.005). No statistical differences were recorded in terms of estimated blood loss (*P* value 0.05), length of hospital stay (*P* value 0.74), complications (*P*‐value 0.31), and postoperative pain (*P* value 0.06 at 2 h postoperatively). Cosmetic outcomes acquired through subjective assessment by the patient and surgeon at discharge and 1 month after surgery demonstrated a statistically significant higher satisfaction rate in the MiniLap compared to the S‐LPS group.

**Conclusion:**

Our study demonstrates that salpingo‐oophorectomy performed with the MiniLap system is feasible, safe, and well‐tolerated by patients. Furthermore, this technique has proven to be non‐inferior to standard LPS in terms of postoperative pain, blood loss, hospital stay duration, and complications.

## 1 INTRODUCTION

Surgery is a medical procedure that involves the incision of body tissues to treat or diagnose a disease. Surgery causes tissue trauma and the release of inflammation and pain mediators.^1^ Postoperative pain is an expected but undesirable outcome of surgical procedures.^2^ Effective post‐surgical pain management has been associated with patient satisfaction, early mobilization, reduced hospital stay, and cost savings.^3^ For these reasons, routine surgical practice should prioritize surgical techniques to reduce postoperative pain. In women undergoing bilateral salpingo‐oophorectomy surgery, more studies have demonstrated the superiority of laparoscopy over laparotomy in reducing morbidity, urinary tract infections, postoperative complications, postoperative pain, length of hospital stay, and total cost.^4^, ^5^ The primary advantage of minimally invasive procedures is the absence of a large abdominal incision, which results in fewer wound‐related complications and a reduction in postoperative pain.^6^ In recent years, significant advancements in minimally invasive surgery have been seen to reduce the morbidity associated with laparoscopic surgery, focusing on reducing the number or size of instruments.^7^ In this context, it is possible to consider using percutaneous instruments (MiniLap percutaneous surgical system [PSS]).^8^ They are characterized by instruments with a maximum diameter of 2.4 mm, without applying trocars. Considering the importance of reducing postoperative pain and the evolution towards less invasive surgical techniques, we performed a retrospective case–control study comparing the MiniLap PSS versus standard laparoscopy in salpingo‐oophorectomy surgeries, aiming to demonstrate the non‐inferiority of this ultra‐minimally invasive surgical technique concerning the current gold standard.

## 2 MATERIALS AND METHODS

This was a retrospective, single‐center propensity‐matched case–control study conducted at Udine's Department of Gynecology and Obstetrics of University Hospital. We enrolled all patients on whom a salpingo‐oophorectomy was performed between January 2022 and December 2023. Inclusion criteria were patients over 14 years of age, undergoing salpingo‐oophorectomy surgery for benign pathology; ASA <3. Exclusion criteria were patients who had undergone other surgery during salpingo‐oophorectomy, diagnosed or suspected of gynecologic malignancies, and ovarian masses larger than 10 cm due to a high risk of cyst rupture. Institutional review board approval was obtained (Institutional Review Board—Department of Medicine, University of Udine, IRB‐DMED. Prot IRB 182/2022), and before recruitment, patients involved in the study gave their written informed consent to participate. Clinical data regarding the population study were retrieved from the hospital management system and recorded in an anonymous database. Highly experienced gynecologic endoscopic surgeons (>500 gynecologic surgical procedures) conducted all surgeries. The operative time (OT) was between the skin incision and closure. Postoperative complications were assessed within the first 30 days following the procedure using the Clavien‐Dindo classification. The intraoperative anesthesiology protocol was strictly standardized and followed in all cases. Anesthesia was induced with a bolus of propofol 1.5/2 mg/kg followed by the administration of fentanyl 5–6 mg/kg; vecuronium bromide 0.1 mg/kg was used to facilitate tracheal intubation and obtain muscle relaxation. General anesthesia was maintained with sevoflorane 1.5%–2% end‐tidal concentrations. Sevoflurane was tailored to maintain the bispectral index values between 35 and 40. Patients were mechanically ventilated with 50% oxygen in air, with the minute ventilation adjusted to maintain end‐tidal CO_2_ concentrations between 30 and 32 mmHg. Paracetamol 1000 mg intravenously at the beginning of the surgical procedure and ketoralac 30 mg intravenously at the end of surgery were administered for postoperative pain control. Metoclopramide 10 mg and ranitidine 1 mg/kg were given intravenously before surgery; ondansetron hydrochloride 8 mg was administered 30 min before the expected time of extubation. To correctly assess pain related to the abdominal incision, neither pre‐ nor postoperative local anesthesia at the level of each incisional post was used. No other analgesic drug, intravenous or intramuscular, was given to the patients, except for those directly required by the patient herself, which were meticulously registered (paracetamol 1000 mg orally/intravenously). Postoperative pain assessment during the immediate postoperative period was performed in all patients using a validated visual analog scale (VAS), scored from 0 to 10 (0 = no pain; 10 = unbearable pain). Postoperative pain was subjectively reported 2, 4, 12, and 24 h after surgery. Postoperative pain management was the same for all patients, involving the administration of paracetamol 1 g every 8 h for 72 h and ketorolac 30 mg every 8 h for 48 h. At the time of discharge and after 1 month aesthetic outcomes were assessed through a questionnaire administered to both the surgeon and the patient. Both rated the abdominal scars using a numerical rating scale (NRS) from 0 to 10. Due to the different types of scars and ethical considerations regarding transparency about the performed surgery, both were aware of the surgical technique used at the time of evaluation.

### 2.1 Surgical techniques

**FIGURE 1:**
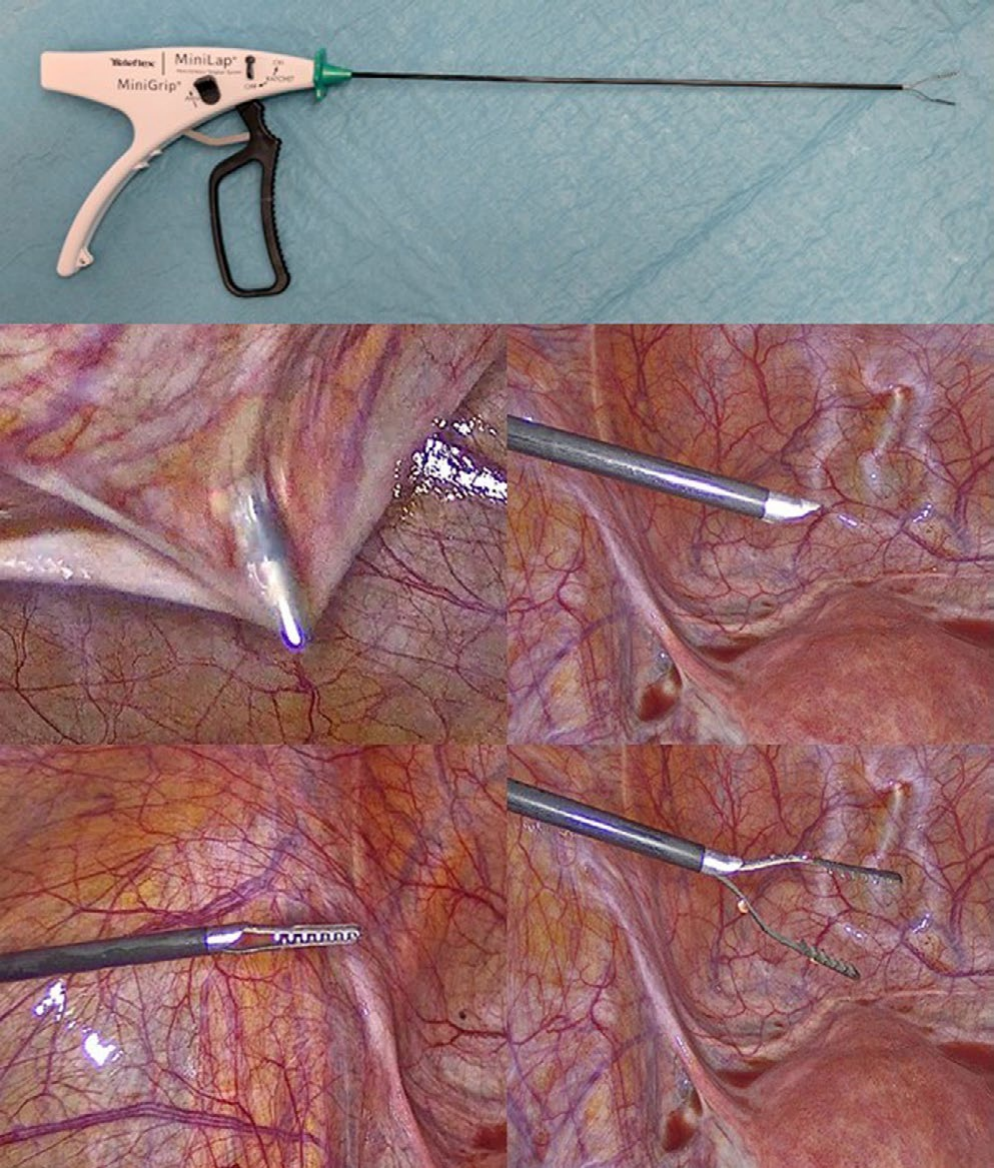
MiniLap percutaneous surgical system.

The difference between the two surgical methods lies in the type of two laparoscopic access. In both standard laparoscopy (S‐LPS) and the MiniLap percutaneous surgical system (PSS) (MiniLap PSS, Teleflex Inc., USA), the initial laparoscopic access was performed at the umbilical level using open laparoscopy (Hasson technique). The subsequent two accesses for both methods were carried out in the right and left iliac fossa. In the PSS, these latter accesses are directly performed using MiniLap with a 2.4 mm incision, while in standard laparoscopy, they are performed using 5 mm trocars. The percutaneous grasping forceps features a sharp tip when closed, enabling system insertion through the skin without a scalpel. Once inside the abdomen, a grasping instrument is deployed and ready for use (Figure 1). Finally, in both techniques, a 5 mm ancillary trocar was placed in the suprapubic region to use instruments such as bipolar forceps, irrigators, and scissors. Multifunction instruments for these surgical procedures were not required. The subsequent surgical steps were identical for both techniques. In both cohorts of patients, at the end of the surgical procedure, fascia at the umbilical trocar site was closed with a 0‐Vicryl, and a 3/0 reabsorbed Vicryl suture was used for the skin closure. A syringe meticulously measured estimated blood loss before final pelvic washing.

### 2.2 Statistical analysis

The primary endpoint of our study was to assess postoperative pain following surgical treatment with MiniLap compared to standard laparoscopy (S‐LPS), using subjective pain assessment. As secondary endpoints, we evaluated differences in surgical duration, blood loss, postoperative complications within 30 days of the procedure, and cosmetic results at 30 days after surgery. However, we performed a propensity‐matched analysis because of the nonrandomized nature of the study design and the potential allocation biases rising from the retrospective comparison between the groups (Minilap vs. S‐LPS). Propensity matched comparison attempts to estimate the effect of a treatment by accounting for possible factors (e.g., constitutional variables) that predict receiving the treatment. Propensity matched comparison aims to reduce biases arising from different covariates. A propensity score was developed through a multivariable logistic regression model: age, body mass index (BMI, calculated as weight in kilograms divided by the square of height in meters; less or more than 25 kg/m^2^), previous surgery. Patients undergoing Minilap were matched 1:1 to patients undergoing S‐LPS using a caliper width <0.1 standard deviations of the estimated propensity score's logit odds. The sample size after matching was *N* = 80 (*N*_cases_ = 40; *N*_controls_ = 40). This dimension allowed us to detect, with a power = 80%, an expected proportion of pain >8 of 50% (controls group) and 22% (cases group) with two‐side *α* = 0.05. A detailed description of propensity matching is described elsewhere.^9^, ^10^, ^11^, ^12^, ^13^, ^14^ Descriptive statistics were employed to characterize patients and surgical features. Quantitative variables are described using mean or median and interquartile range (IQR) for non‐normally distributed variables. Statistical analysis was performed using Pearson's chi‐square test, while Fisher exact test was used to compare results with rates ≤0.1%. A significance level of *P* less than 0.05 was set to determine statistical significance in each analysis. The SPSS (version 17.0, SPSS Inc., Chicago, Illinois) and NCSS statistical software (version 11.0, NCSS statistical software, Kaysville, Utah) were used.

## 3 RESULTS

**FIGURE 2:**
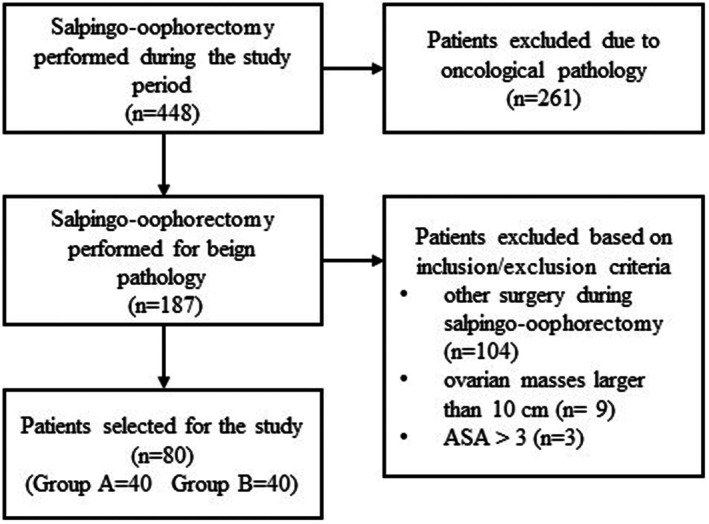
Study design and selection process.

During the study period, 187 women underwent salpingo‐oophorectomy for benign gynecologic disease at the Clinic of Obstetrics and Gynecology, Santa Maria della Misericordia University Hospital, Udine, Italy. A total of 80 surgical patients were selected, with 40 in each group (MiniLap in group A and S‐LPS in group B). Figure 2 describes the study design and selection process. The average age was 50.7 years (14–77) and 52.9 years (21–78) for groups A and B, respectively. No significant differences in the baseline characteristics were observed between the two groups. The median BMI was 23.1 kg/m^2^ (range, 18.0–24.0) for group A and 23.9 (range, 16.0–34.0) for group B. The demographic data are summarized in Table 1.

**TABLE 1:** Demographic data according to different types of surgery.

Variables	Group A (MiniLap)	Group B (S‐LPS)	*P* value
No. of patients	40	40	/
Median age (years)	50.7 (14–77)	52.9 (21–78)	0.521
Median BMI (kg/m^2^)	23.1 (18–34)	23.9 (16–34)	0.513
Previous abdominal surgery (*n*) (%)	16 (40%)	18 (45%)	0.652
Type of disease (*n*) (%) Adnexal cyst Prophylactic surgery	28 (70%) 12 (30%)	35 (87.5%) 5 (12.5%)	0.056

*Note*: BMI, calculated as weight in kilograms divided by the square of height in meters.

Abbreviation: BMI, body mass index.

The median operative time for the entire procedure was 57.7 min (range, 28.0–125.0) in group A and 75.5 min (range, 22.0–180.0) in group B, with significant statistical differences in the two groups (*P* value 0.005).

**FIGURE 3:**
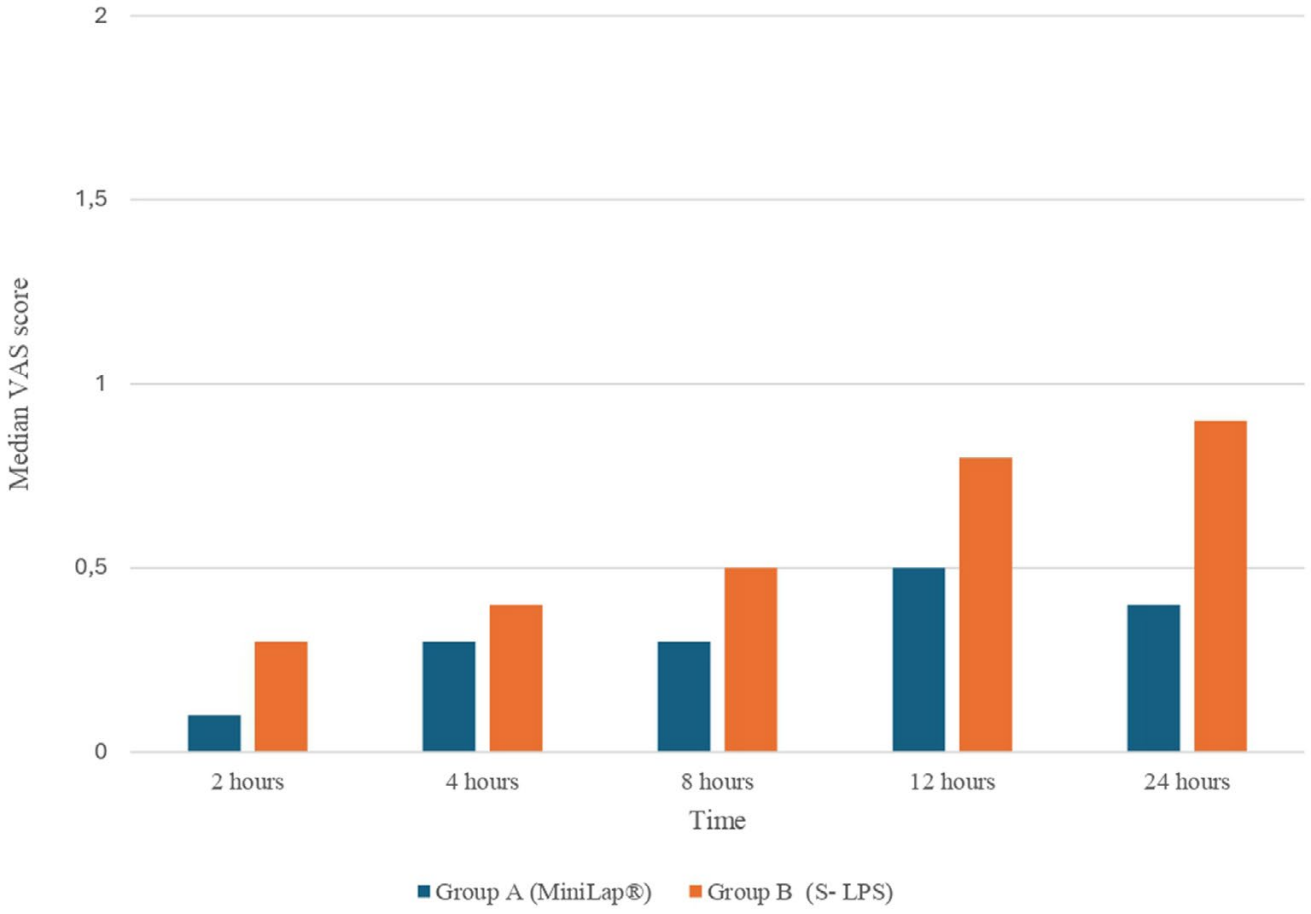
Pain assessment by visual analog scale (VAS).

**FIGURE 4:**
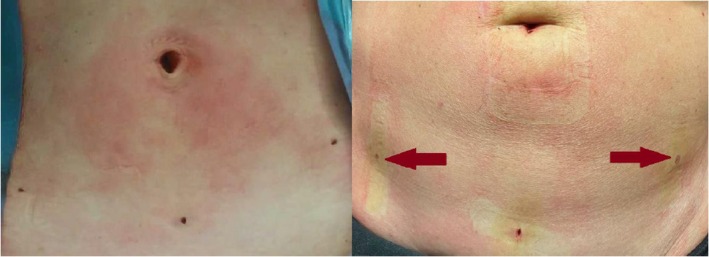
Cosmetic outcomes: comparison of incisional scars at the end of surgical procedure and 3 days after surgery.

No statistical differences were recorded in terms of median estimated blood loss (EBL), <50 mL range (0–200) in both groups; the median length of hospital stay 1.3 days (1–5) in group A and 1.8 days (1–9) in group B (*P* value 0.742); the median postoperative pain for group A was 0.1 on the NRS scale (0–5), while for group B, it was 0.3 (0–2), with a *P* value of 0.062, at 2 h postoperatively. Figure 3 present the data for postoperative pain. According to the Dindo classification, we observed only one case of postoperative complications (grade 2) in the S‐LPS group: a patient who underwent a transfusion due to postoperative anemia. The results are summarized in Table 2. Table 3 summarizes the cosmetic results. We observed a statistically significant higher satisfaction rate in the MiniLap than in the S‐LPS group, according to both the patient and the surgeon at each evaluation time. Figure 4 shows the cosmetic results obtained.

**TABLE 2:** Perioperative data according to different types of surgery.

	Group A (MiniLap)	Group B (S‐LPS)	*P* value
Median OT (range); minutes	57.7 (28–125)	75.5 (22–180)	**0.005**
Median EBL (range); mL	<50 (0–200)	<50 (0–200)	0.051
No. of intraoperative complications (%)	0	1	0.312
Median discharge (days)	1.3 (1–5)	1.8 (1–9)	0.742
Median NRS score (range)			
2 h	0.1 (0–2)	0.3 (0–5)	0.062
4 h	0.3 (0–2)	0.4 (0–4)	0.571
8 h	0.3 (0–4)	0.5 (0–4)	0.442
12 h	0.5 (0–3)	0.8 (0–4)	0.362
24 h	0.4 (0–2)	0.9 (0–4)	0.401

Abbreviations: EBL, estimated blood loss; NRS, numerical rating scale; OT, operative time.

**TABLE 3:** Cosmetic results (visual analog scale, mean [SD]).

Time	Patient			Surgeon		
	MiniLap	S‐LPS	*P* value	MiniLap	S‐LPS	*P* value
Discharge	9.35 (1.03)	7.48 (1.21)	0.001	9.47 (0.59)	7.86 (0.76)	0.001
Postoperative day No. 30	9.56 (0.83)	8.43 (0.76)	0.008	9.85 (0.71)	8.26 (0.87)	0.02

## 4 DISCUSSION

Few studies have been published regarding MiniLap treatment of adnexal disease,^8^, ^15^, ^16^, ^17^ as shown in Table S1. These are mainly case reports or pilot studies demonstrating the feasibility of this technique in a pretty significant group of patients. In contrast, no data are available for comparison between MiniLap and S‐LPS approaches in gynecologic surgery, even more if we consider the postoperative pain data. Indeed, the present study, as well as underlining that percutaneous surgery with the MiniLap system is a feasible and safe procedure, to our knowledge is the first propensity‐match case–control on MiniLap versus S‐LPS which demonstrates no significant differences in complications, estimated blood loss, postoperative hospitalization, and postoperative pain between the two approaches. However, other studies performed with percutaneous systems have obtained similar results. Indeed, even when the differences in postoperative pain and requirement of analgesic drugs between the two groups were recorded as statistically significant in the literature, they might appear to be of low clinical relevance because they do not reflect a different length of stay for the two approaches. A possible explanation to justify our data is the presence of painful lower/mid‐quadrant incisions through the rectus musculature. Chou et al.^18^ described less postoperative pain on day 0 using a transumbilical port than a transabdominal one for the specimen's retrieval, independently from other additional ports. However, some evidence demonstrates that reducing the port diameters provides an advantage regarding postoperative pain without impairing the ability to accomplish the procedure safely.^9^, ^15^

In the study conducted by Rossitto et al., various minimally invasive techniques (single‐site surgery, 3 mm laparoscopy, and percutaneous system) were compared with standard laparoscopy in 100 patients divided into four groups in a hysterectomy procedure.^19^ The PSS group reported the shortest surgical time compared to all the surgical techniques. This data could be attributed to using a multifunctional tool in PSS procedures, compensating for the lack of energy in percutaneous devices and reducing the need for changing instruments typically required during a surgical procedure.^19^

In our case series, the only significant differences between MiniLap and S‐LPS were the operative time and cosmetic results (Tables 2 and 3). This can be explained by the fact that in PSS with MiniLap, inserting additional trocars in the right and left iliac fossa is unnecessary, thus reducing the surgical time. Additionally, due to the small size of the incisions, wound closure was performed at the end of the procedure using adhesive strips without the need for sutures. We recorded only one postoperative complication in a patient undergoing standard LPS surgery. We should consider that we enrolled only 40 cases, but no patients who underwent surgery with the Minilap system had complications 30 days after surgery. Other studies have evaluated perioperative outcomes of minimally invasive techniques, obtaining comparable results.^20^, ^21^, ^22^, ^23^, ^24^, ^25^

Our data showed that satisfaction in terms of aesthetic results was higher in the group undergoing surgery with MiniLap system than in the S‐LPS group. This is especially important for prophylactic surgery since these women are usually young and undergo surgery to remove potentially healthy organs. These results are in line with those obtained from other studies performed on PSS.^26^, ^27^ In addition, Fanfani et al. reported that improved cosmetic results obtained through a minimally invasive approach also enhanced physical and psychological well‐being.^28^

The current trend in endoscopy is to increasingly minimize invasiveness while ensuring safety and surgical efficacy, optimizing outcomes and improving cosmetic results.

The limitations of the 3 mm devices in tissue manipulation are overcome by using a standard laparoscopic instrument, used through a 5 mm suprapubic trocar, mimicking the standard laparoscopic environment in terms of instrument triangulation and use.^16^, ^29^, ^30^, ^31^ In our study, we also inserted a 5 mm trocar at the suprapubic level to enable the use of electrified instruments. The absence of electricity is currently one of the primary limitations of these devices.

Currently, the MiniLap system is more expensive than standard laparoscopy, partly because it is a single‐use instrument. However, this cost difference is partially balanced by the shorter procedure duration, the elimination of two trocars, and the reduced need for sutures. Additionally, greater patient acceptance of the scars associated with the MiniLap system could lead to lower overall societal costs; for example, by reducing the demand for corrective aesthetic treatments or psychological support. In the future, increased competition may help drive down costs, making this technology more accessible.

The principal strength of this trial was the standardization of both (1) intra‐ and postoperative protocols of analgesia, which were strictly controlled and followed in both groups and (2) one protocol for the quantification of postoperative pain, which was carefully standardized by frequently evaluating VAS score (4 times in the first 8 h) at fixed times.

The limitations of the study include its retrospective, non‐randomized design, small sample size, and the heterogeneity of pathologies among patients undergoing salpingo‐oophorectomy, although the propensity score matching between cases and controls should theoretically guarantee the quality of data limiting the affecting variables on the surgical times. Furthermore, MiniLap is in continuous evolution: integrating the system with bipolar grasp could reduce operative time and make this procedure even less invasive in the future.

## 5 CONCLUSION

Our study demonstrates that salpingo‐oophorectomy performed with the MiniLap system is feasible, safe, and well‐tolerated by patients. Furthermore, this technique achieved comparable results to standard LPS in terms of postoperative pain, blood loss, hospital stay duration, and complications. Additionally, the MiniLap group achieved better aesthetic outcomes, which is particularly important in prophylactic surgery. This device allows surgeons to maintain a standard setup, eliminating the need for additional training compared to single‐site surgery. Further studies are necessary to better define the advantages and costs of this new approach compared to other minimally invasive techniques.

## AUTHOR CONTRIBUTIONS

All authors contributed to the study conception and design. Material preparation, data collection and analysis were performed by S.R., G.V., M.A. and F.P. All authors commented on previous versions of the manuscript. All authors read and approved the final manuscript.

## FUNDING INFORMATION

This study received no external funding.

## CONFLICT OF INTEREST STATEMENT

The authors declare no conflict of interest.

## Supporting information


**Table S1.** Published studies on MiniLap treatment of adnexal disease.

## Data Availability

Data is available from the authors upon reasonable request.
