# Supplementary material for: Postoperative pain after MiniLap percutaneous versus standard laparoscopic salpingo‐oophorectomy: A propensity‐matched study
Source: Int J Gynaecol Obstet. 2025 Mar 11;170(2):857–64. doi: 10.1002/ijgo.70060 (PMC12255926; doi:10.1002/ijgo.70060)
Supplement: Supplementary file 1 — Table S1. Published studies on MiniLap treatment of adnexal disease. [file IJGO-170-857-s001.docx]

**Supplemental Table 1. Published studies on MiniLap ® treatment of adnexal disease.**

| **Author** | **Title** | **Type of study** | **N° of cases** | **Disease** | **Type of surgery** | **Results** |
| --- | --- | --- | --- | --- | --- | --- |
| S. Restaino et al. | Scar-Free Laparoscopy in BRCA-Mutated Women | Case report | 1 | BRCA1 mutation | Bilateral salpingo-oophorectomy | OT, EBL consistent with the standard. No intra and postoperative complications. |
| A. Rosati et al. | Needleoscopic-assisted risk-reducing bilateral salpingo-oophorectomy in BRCA1/2 mutation carriers: Peri-operative outcomes and psychological impact | Prospective | 26 | BRCA1/2 mutation | Bilateral salpingo-oophorectomy | Decreased postoperative incisional pain, reduced hospitalization, and improved aesthetic and psychological outcomes. |
| S. Gueli Aletti et al. | Needleoscopic Conservative Staging of Borderline Ovarian Tumor | Case report | 1 | Borderline ovarian tumor | Right ovarian cystectomy, peritoneal biopsies, infracolic omental biopsy, and peritoneal cytology | OT, EBL consistent with the standard. No intraoperative complications. |
| A. Boza et al. | Mini-Laparoscopic Gynecological Surgery Using Smaller Ports Minimizes Incisional Pain and Postoperative Scar Size: A Paired Sample Analysis | Prospective | 110 | Benign gynecological disease | 61 hysterectomy ± salpingo-ophorectomy, 28 ovarian cystectomy, 18 adnexal surgery, 3 myomectomy | Lower pain scores for 2.4 and 3 mm ports than those for 5 mm ports. Two port-related complications occurred: one subcutaneous emphysema and one bleeding from a 5-mm trocar site. |
